# Supplementary material for: Imaging Findings in Patients with Immune Checkpoint Inhibitor-Induced Arthritis
Source: Diagnostics (Basel). 2022 Aug 13;12(8):1961. doi: 10.3390/diagnostics12081961 (PMC9406920; doi:10.3390/diagnostics12081961)
Supplement: Supplementary file 1 [file diagnostics-12-01961-s001.zip › diagnostics-1819894-SI.pdf]

Supplementary Table S1 General characteristics of patients with ICI-induced arthritis with MRI assessment.

Author [Ref]	Gender	Underlying disease	Preexisting joint disease	Type of CPI	Clinical presentation	MRI Findings
Subedi A [34]	Male	Kaposi sarcoma	Tendinopathy	Pembrolizumab	Inflammatory arthritis	Hand: tenosynovitis of bilateral flexor tendons
Subedi A [34]	Male	Thyroid cancer	Tendinopathy	Pembrolizumab	Inflammatory arthritis	Hand: Synovitis, marginal erosions and tenosynovitis of the extensor and flexor, periarticular marginal bone edema in carpal and MCP
Subedi A [34]	Female	Urothelial carcinoma	OA	Nivolumab	Inflammatory arthritis	Hand: tenosynovitis of extensors
Subedi A [34]	Female	Cervical cancer	None	Nivolumab	Inflammatory arthritis	Hand: Multifocal osseous erosions in distal radius, distal ulna, carpal and MCP bones, synovitis intercarpal joints and tenosynovitis
Subedi A [34]	Female	Colon cancer		Avelumab	Inflammatory arthritis	Hand: no erosions, tenosynovitis or synovitis
Subedi A [34]	Female	Pheochromocytoma	None	Nivolumab and ipilimumab	Inflammatory arthritis	Knee: Moderate effusion and diffuse thickening of synovium
Subedi A [34]	Female	Cervical cancer	None	Bintrafusp alfa	Inflammatory arthritis	Ankle: joint effusion, thickening of tibiotalar joint synovium, peroneal tenosynovitis
Subedi A [34]	Female	Lung cancer	RA	Bintrafusp alfa	Inflammatory arthritis	Hand: Erosions at the radial styloid
Cappelli LC [16]	Male	Renal Cell Carcinoma	NA	Nivolumab and ipilimumab	Inflammatory arthritis	Ankle: tibiotalar and subtalar joint effusions with marked synovitis
Feist J [35]	Male	Melanoma	None	Nivolumab	SpA-Like	Spine: cervical and thoracic spine showing widespread contrast uptake in the sternocostal joint (arrow in facet joints and costovertebral joints
Kobak S [36]	Male	Lung adenocarcinoma	NA	Pembrolizumab	Oligoarthritis	Leg: Diffuse edema in both gastrocnemius muscle and fascia, and abnormal facial signal intensity and enhancement
Daoussis D [24]	10 cases 8 Male	Lung carcinoma (n=4) Bladder (n=3) Renal cancer (n=2) Melanoma (n=1)	NA	Nivolumab (n=7) Pembrolizumab (n=1) Durvalumab (n=1) Atezolizumab (n=1)	-Prominent joint involvement (n=3) -Prominent ‘periarticular’ involvement (n=4) -Myofasciitis (n=3)	-Joint pattern: synovitis and fasciitis around the involved joints -Periarticular pattern: tenosynovitis with more prominent myositis and/or fasciitis in the surrounding tissues -Myofascitis pattern: myofasciitis of the surrounding muscles
Current series Case 1	Male	Acute myeloid leukemia	Uveitis HLA-B27 positive	Anti-TIM3	Monoarthritis	Sacroiliac: No erosions, no bone edema or signs of sacroiliitis
Current series Case 2	Female	Melanoma	None	Pembrolizumab	PsA-like	Hand: Bone marrow edema from second to fifth fingers in distal phalanges
Current series Case 3	Male	Chronic myeloid leukemia	None	Anti-TIM3	Tenosynovitis	Ankle: Tenosynovitis in common extensors
Current series Case 4	Male	Liver carcinoma	None	Durvalumab	Inflammatory arthritis	Whole body MRI: Glenohumeral synovitis and knee synovitis

Anti-TIM3: Anti T cell immunoglobulin mucin domain 3; NA: Not available; OA: Osteoarthritis; RA: Rheumatoid arthritis; SpA: Spondyloarthritis.

Supplementary Table S2 General characteristics of patients with ICI-induced arthritis with PET/CT scan assessment.

Author [Ref]	Gender	Underlying disease	Preexisting joint disease	Type of CPI	Clinical presentation	PET-CT Findings
Schierz JH [25]	Male	Melanoma	NA	1. Nivolumab<br>2. Pembrolizumab	Arthritis and swollen arms and legs muscles	1. Mildly increased uptake in the left knee and, bilaterally, in the mediastinal and hilar lymph nodes (sarcoid like)<br>2. Diffusely increased metabolic activity in the muscles (correlated with clinical findings of swollen arms and legs). Also showed a slight progression in left knee uptake, mildly increased uptake in the right knee and ankle.
Schierz JH [25]	-	Melanoma	NA	Nivolumab	PMR-like	Before treatment, diffusely increased uptake in the knees. After treatment, increased uptake in the interspinous bursae of the lumbar spine, highly suggestive of PMR, and a further increase in uptake in the knees.
Leipe J [37]	Female	Melanoma	NA	Nivolumab and Ipilimumab	Inflammatory arthritis (RFA and anti-CCP positive) and PMR	NA
Leipe J [37]	Male	Non-small cell lung cancer	NA	Nivolumab	Seronegative monoarthritis	NA
Leipe J [37]	Male	Melanoma	NA (Other irAEs: pancreatitis, pruritus)	Ipilimumab and Pembrolizumab	Seronegative monoarthritis	NA
Leipe J [37]	Male	Melanoma	NA (Other irAEs: Colitis, myasthenic symptoms)	Pembrolizumab	Oligoathritis and myositis seronegative	NA
Leipe J [37]	Male	Non-small cell lung cancer	NA	Nivolumab	Oligoarthritis RF positive, anti-CCP negative	NA
Nobashi T[41]	-	Melanoma	NA	Pembrolizumab	Intermittent joint and muscle pain	Symmetric FDG uptake in multiple joints: shoulder, elbows, wrist, hips, and knees and lymph nodes.
Maniu C [39]	Male	Melanoma	NA	Ipilimumab	Progressive joint and muscular pain, fever, an RFA elevated	enhanced FDG uptake in the muscle insertion of the extremities (shoulder, elbows, wrist, hips, and knees)
Nobashi T [27]	5 Male and 1 female	Melanoma	NA	Pembrolizumab	Arthritis	NA
Nobashi T [38]	Male	Melanoma	NA	Ipilimumab	Asymptomatic	Diffuse FDG avidity of multiple joints.
Bronstein Y [27]	Female	Melanoma	NA	Ipilimumab	Inflammatory arthralgia and myalgia in both thighs	symmetric increased FDG uptake in multiple joints and bilateral symmetric mildly FDG-avid hilar lymph nodes.
Bronstein Y [27]	Female	Melanoma	NA	Ipilimumab	Asymptomatic	FDG avidity sacroiliac synovitis
Bronstein Y [27]	Male	Melanoma	NA	Tremelimumab	Asymptomatic	Bilateral symmetric FDG avid axillary lymphadenopathy,

						bilateral synovial effusion, and FDG avidity in shoulder joints.
Gauci ML [40]	Male	Melanoma	NA	Nivolumab	RS3PE	PET preformed 6 and 3 before the symptoms had already shown isolated hypermetabolism of the shoulder girdle and wrist.
Van Der Geest KSM [26]	5 cases	Bladder, squamous-lung, Lung adeno., Renal cancer	NA	Pebrolizumab or Nivolumab	PMR	FDG uptake at the shoulders, hip joints, greater trochanter, ischial tuberosities, sternoclavicular joints and cervical/lumbar interspinous bursae. Four patients underwent PET-CT prior to ICI therapy. These scans showed a grade 1-2 FDG uptake at the shoulders and hips.
Current series	Male	Melanoma	None	Nivolumab	PMR	Symmetric FDG uptake shoulder, hip joints, greater trochanter and ischial tuberosities.
Current series	Male	Prostate	None	Atezolizumab and Cabozatinib	PMR	Symmetric FDG uptake shoulder, hip joints, greater trochanter and ischial tuberosities. Also showed a FDG uptake in left knee uptake. After discontinuation of the treatment, decreased the uptake (figure 4).

NA: Not available; PMR: Polymyalgia rheumatica; RS3PE: Remitting Seronegative Symmetrical Synovitis with Pitting Edema.
